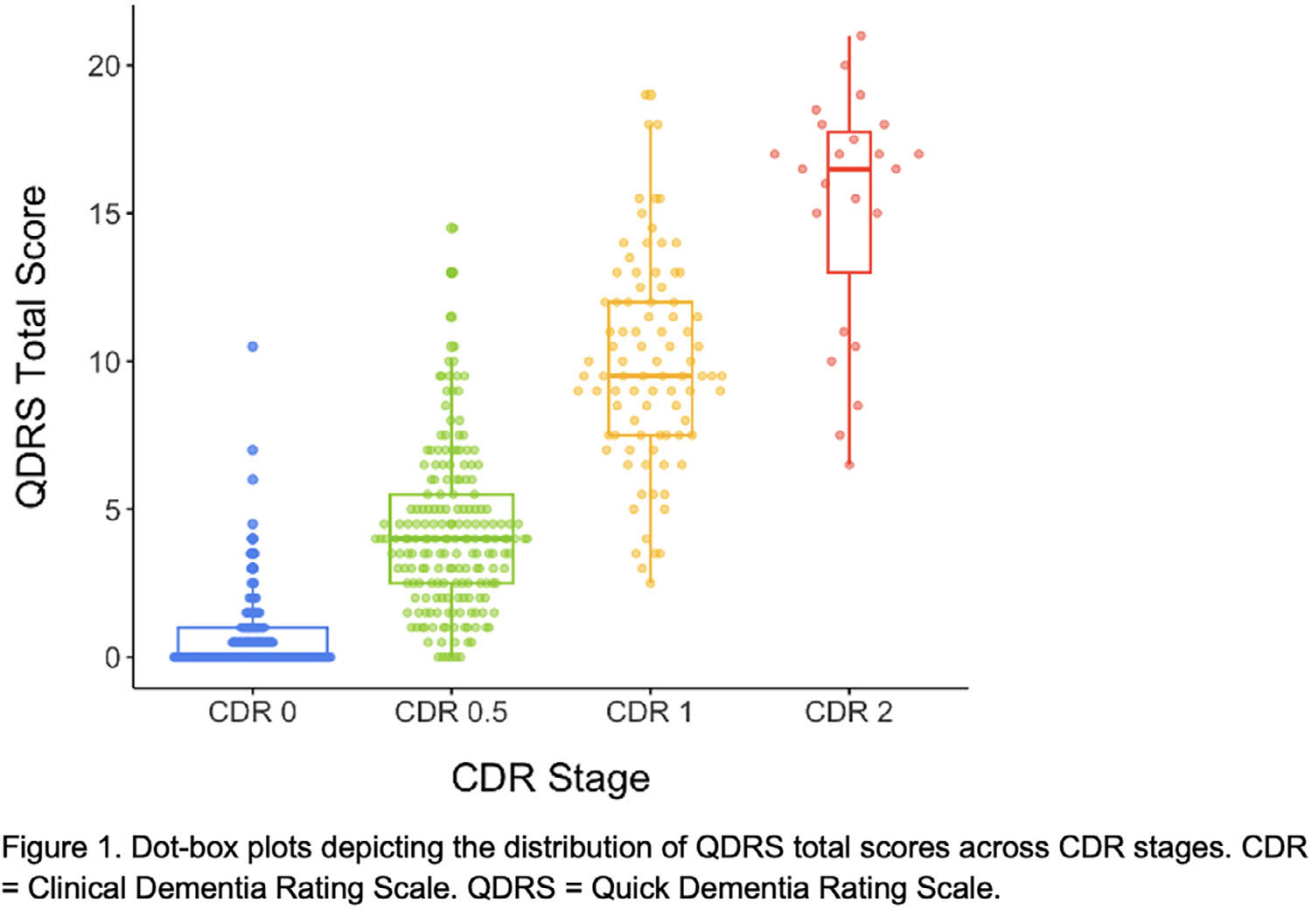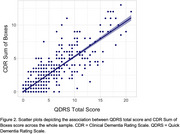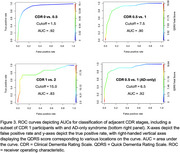# Identification of Optimal Cutoff Scores for the Quick Dementia Rating Scale as a Brief, Self‐Administered Alternative to the Clinical Dementia Rating Scale

**DOI:** 10.1002/alz70857_104681

**Published:** 2025-12-25

**Authors:** Alexandra J. Weigand, Tiffany R. Brailow, Sabrina Jarrott, Kelly J. Atkins, Claudio Reck‐Rivera, Elena Tsoy, Katherine L. Possin

**Affiliations:** ^1^ Memory and Aging Center, University of California San Francisco, San Francisco, CA, USA

## Abstract

**Background:**

There is a need for brief and scalable measures of dementia severity to determine treatment eligibility and inform clinical decision‐making. We assessed the psychometric properties and optimal cutoff scores of the Quick Dementia Rating Scale (QDRS) to predict Clinical Dementia Rating Scale (CDR) stages in a large, well‐characterized, and clinically diverse sample of older adults with and without neurodegenerative conditions.

**Method:**

The 10‐item informant‐reported QDRS was obtained for 902 participants through the University of California San Francisco Alzheimer's Disease Research Center, 610 of whom had concurrent CDR data and 36 of whom had repeat QDRS data within 6 months. We assessed optimal cut‐points based on our sample using receiver operating characteristic (ROC) analyses including bootstrapped comparison of area under the curve (AUC) against previously published QDRS cut‐points that were based on 267 participants. Reliability (internal, test‐retest) and validity (criterion, convergent, divergent) of the QDRS were also assessed.

**Result:**

The QDRS correlated strongly with the CDR sum of boxes (ρ = .87, p < .001, Figure 1) and significantly differed across CDR stages (F = 548.0, p < .001, Figure 2). It also had strong internal reliability (Cronbach's alpha = .94) and acceptable test‐retest reliability (ρ = .57). Our optimal cut‐point replicated the published cutoff of 1.5 (AUC = .92, Figure 3) to differentiate CDR 0 vs. 0.5 (AUC = .85). When differentiating CDR 0.5 vs. 1, our optimal cutoff was slightly higher (7.5 with AUC = .90 vs. 6.0 with AUC = .84) although when we limited out CDR 1 sample to individuals with an Alzheimer's disease clinical syndrome, a more similar cut‐point of 6.5 was found (AUC = .91, Figure 3). Recommendations for adjusting the cut‐point for less typical neurodegenerative presentations (e.g., linguistic, behavioral, motor predominant) will be presented at AAIC 2025.

**Conclusion:**

The QDRS offers an efficient and scalable measure of dementia severity that can be used as a reliable and valid clinical alternative to the CDR, including for potential determination of eligibility and clinical monitoring for emerging AD treatments.